# Endoscopic and pathohistologic features of early gastric signet ring cell carcinoma presented as elevated type: A case report

**DOI:** 10.3389/fonc.2022.1015989

**Published:** 2022-12-08

**Authors:** Lianjun Di, Xinglong Wu, Biguang Tuo

**Affiliations:** ^1^ Department of Gastroenterology, Digestive Disease Hospital, Affiliated Hospital of Zunyi Medical University, Zunyi, China; ^2^ Department of Pathology, Affiliated Hospital of Zunyi Medical University, Zunyi, China

**Keywords:** case report, early diagnosis, endoscopic feature, gastric cancer, signet ring cell carcinoma

## Abstract

**Background:**

Almost all early gastric signet ring cell carcinomas (SRCCs) are the flat or depressed type, and the elevated type is rare. Here, we report the endoscopic and pathohistologic features of a rare case of SRCCs presented as the elevated type.

**Case presentation:**

A 54-year-old man underwent esophagogastroduodenoscopy in our hospital because of intermittent upper abdominal pain for 6 years. White light endoscopy revealed an elevated lesion that is smooth and reddish and covered with normal mucosa and looked like a polyp. Magnifying endoscopy with narrow-band imaging showed broadened intervening parts, an elongated pit, and a dense microvascular network with focal irregularity. The lesion was considered as early gastric cancer and completely resected with endoscopic submucosal dissection. Pathohistological examination confirmed that the lesion was pure early SRCC that was limited within the mucosal lamina propria (T1a).

**Conclusion:**

Elevated pure gastric SRCC is rare. This is a report of early pure gastric SRCC presented as the elevated type and the description of its endoscopic and pathohistologic features, which will contribute to the early detection of gastric SRCC.

## Introduction

Gastric cancer is the fifth most frequently diagnosed cancer and the third leading cause of cancer-related death in the world (1). Gastric signet ring cell carcinoma (SRCC) is a histotype of gastric cancer, which is defined according to the WHO’s classification as a poorly cohesive carcinoma composed mainly of tumor cells with a signet-ring morphology (2). Despite a decrease in the global overall incidence of gastric cancer in recent decades, the incidence of gastric SRCC is continually increasing (3). Previous studies showed that gastric SRCC portends a poor prognosis (4). However, with the advancement of diagnosis and treatment for early gastric cancer, the studies have demonstrated that the prognosis of SRCC at an early stage is better than other types of gastric cancer, while that of SRCC at an advanced stage is relatively poorer (5). The early detection of SRCC, therefore, is important for the improvement of a patient’s prognosis. Early gastric cancer is mainly detected by endoscopy. Almost all gastric SRCCs are observed *via* endoscopy as flat or depressed lesions, and the elevated type is rare (6, 7). Here, we report a rare case of pure SRCC presented as an elevated lesion and evaluated the pathohistology of its elevated appearance.

## Case presentation

**Figure 1:**
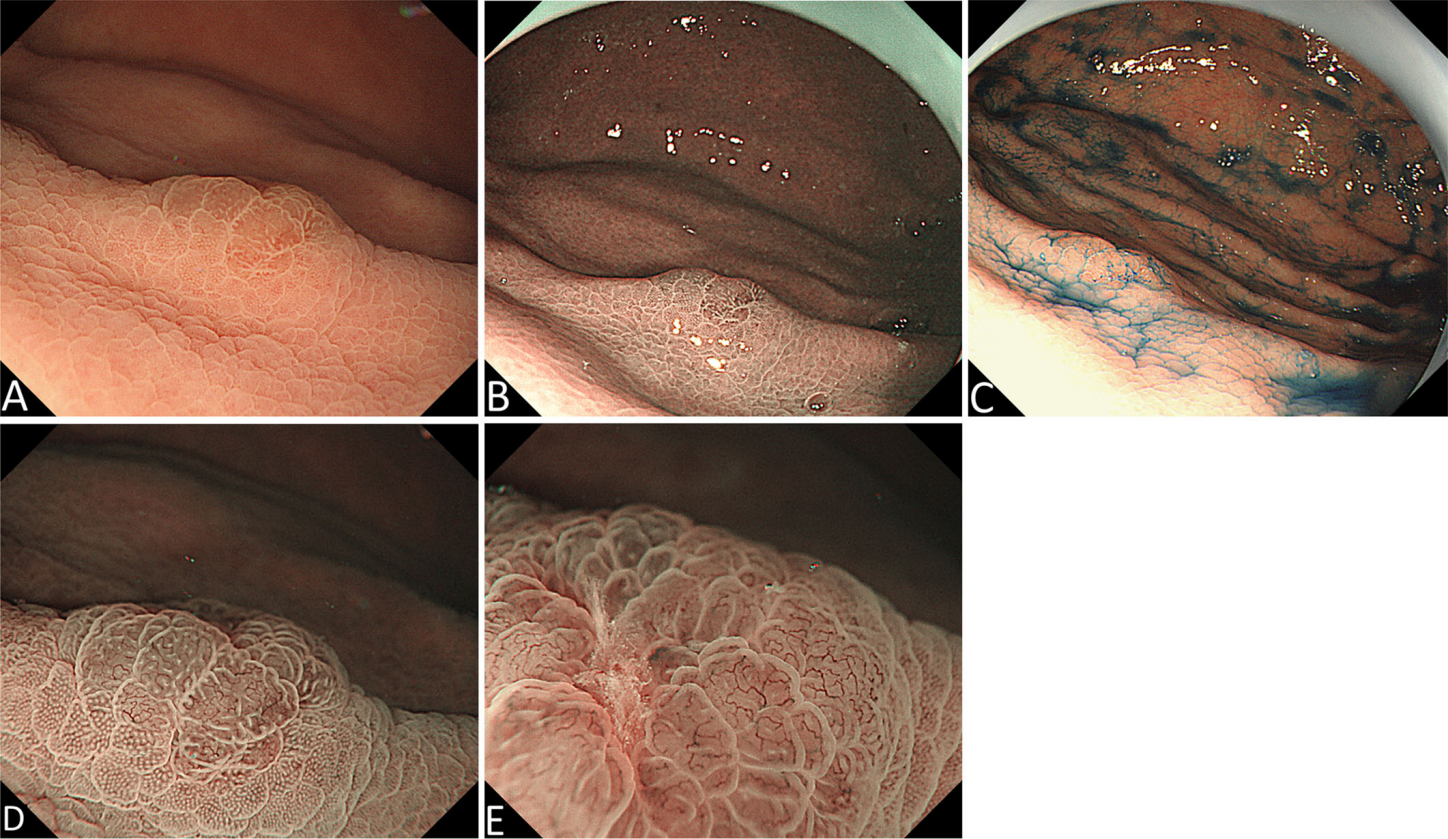
Endoscopic manifestation. **(A)** White light endoscopy (WLE) shows an elevated red and smooth lesion that is covered with normal mucosa and looks like a polyp. **(B)** Endoscopic narrow-band image (NBI) shows an elevated lesion with cyan change. **(C)** Indigo carmine dyeing shows a clear demarcation line and a slightly irregular shape. **(D)** and **(E)** Magnifying endoscopy with NBI shows broadened intervening parts (intercrypt regions), an elongated pit, and a dense microvascular network with focal irregularity.

**Figure 2:**
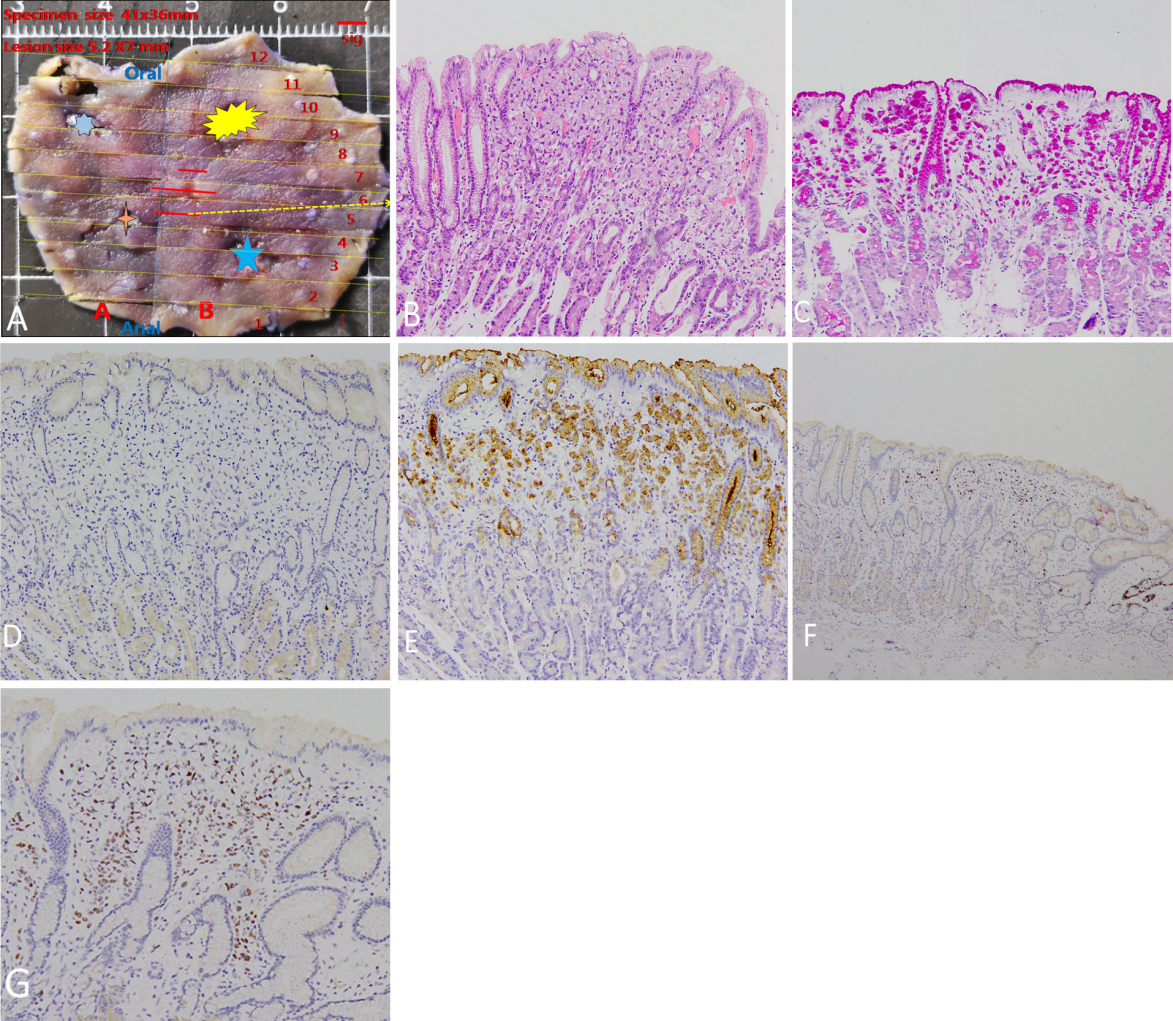
Pathohistologic manifestation. **(A)** The resected specimen. The stars represent the sites of four-quadrant biopsies. The red lines represent lesion areas in each slice. Oral is the oral margin of the specimen. Anal is the anal margin of the specimen. **(B)** Pathohistology shows pure signet ring cell carcinoma. The basement of the lesion is occupied by dense fundus glands, and signet ring cell carcinoma cells grow into the lumen to form an elevated lesion. **(C)** Positive PAS staining. **(D)** Negative MUC-2 staining. **(E)** Diffusely positive MUC-5AC staining. **(F)** Positive Ki-67 staining of 30%. The intense ki67 stain on a deep gland on the far right of the panel is just the proliferative end of the bottom of the crypt. **(G)** Positive P53 staining.

A 54-year-old man underwent esophagogastroduodenoscopy (EGD) in our hospital because of intermittent upper abdominal pain for 6 years with a history of *Helicobacter pylori* infection and eradicating *H. pylori* treatment. The patient had no family history of gastric cancer. The C13 breath test was negative. EGD revealed an elevated lesion with the size of 7 mm at the great curvature of the gastric body, and no other abnormal lesion was detected in the stomach after a meticulous examination. White light endoscopy (WLE) showed that the elevated lesion was smooth and reddish, covered with normal mucosa, and looked like a polyp (**Figure 1A**). The endoscopic narrow-band image (NBI) showed that the elevated lesion presented as cyan change (**Figure 1B**). Indigo carmine dyeing showed a clear demarcation line and a slightly irregular surface pattern (**Figure 1C**). Magnifying endoscopy with NBI (ME-NBI) showed broadened intervening parts (intercrypt regions), an elongated pit, and a dense microvascular network with focal irregularity (**Figures 1D, E**). The widening of the crypt implies that there is some kind of tissue underneath the crypt, such as a tumor, lymphoma, and inflammatory growth that leads to the widening of the crypt. ME-NBI only displayed a dense microvascular network with focal irregularity that is not typical microvascular pattern of early gastric cancer. It is difficult to determine the nature of the lesion under endoscopy; therefore, we performed biopsy, and the examination of biopsy specimens revealed that the lesion was SRCC. We considered the lesion to be early intramucosal gastric cancer. Endoscopic therapy was performed. The lesion was completely resected with endoscopic submucosal dissection. The resected specimen was cut into slices, each at 2 mm width. The red lines represent lesion areas in each slice. Oral is the oral margin of the specimen. Anal is the anal margin of the specimen (**Figure 2A**). Pathohistology showed that the lesion was pure SRCC (pT1a, 0-IIa type, no venous or lymphatic invasions, and negative for horizontal and vertical margins). The basement of the lesion was occupied by dense fundus glands, and the cancer cells grew into the lumen and formed an elevated lesion (**Figure 2B**) with the characteristics of a gastric phenotype. There were positive Periodic acid-schiff (PAS) staining (**Figure 2C**), negative for mucin mucin 2 (MUC-2) (**Figure 2D**), diffusely positive for mucin (MUC)-5AC (**Figure 2E**), the Ki-67 level of 30% (**Figure 2F**), and positive P53 staining (**Figure 2G**). The further cadherin 1 (CDH1) germline mutation test for the patient was negative. Additional surgical resection is not needed, and no recurrence was observed in the 48-month follow-up.

## Discussion

SRCC is a common histologic type of early gastric cancer, and, especially in the *H. pylori–*negative early gastric cancer, most are SRCCs (6, 8). Gastric SRCC usually shows lateral spread and destroys the structure of the stomach glands; therefore, the common endoscopic presentation of early gastric SRRC is a discolored, flat, or depressed lesion and elevated presentation is rare. The majority of elevated-type early gastric cancers is differentiated adenocarcinoma (9). In the previous literature, there were some occasional reports about SRCC presented as an elevated lesion. Most of the elevated gastric SRCC cases reported previously were not a single SRCC and usually composed of SRCC and moderately to poorly differentiated adenocarcinoma (10). The elevated formation was believed to be caused by obvious fibrogenesis or a combination of moderately to poorly differentiated adenocarcinoma, and SRCC produced the elevated lesion. In a case of elevated SRCC recently reported by Misumi et al. (11), there was also an obvious proliferation of fibromuscular tissue in the background mucosa surrounding the lesion that lifted both tumor and non-tumor tissue toward the luminal side to form an elevated appearance. In our case, it was pure SRCC and had no fibrogenesis in the mucosa surrounding the lesion and inside the lesion, which is different from previously reported gastric elevated SRCC. Considering the pathohistological finding in this case with dense fundus glands at the basement of the lesion and remnant glands within the lesion, it can be speculated that dense fundus glands prevent cancer cells from growing downward and remnant glands support cancer cells to grow into the lumen, which leads to the formation of an elevated lesion. Pure gastric SRCC presented as the elevated type is rare. When an elevated lesion was found in the stomach, endoscopists should first differentiate neoplastic from non-neoplastic lesions. It is also important to differentiate epithelial from non-epithelial tumors for a suspected tumor lesion. Endoscopists should not overlook the elevated type of SRCC, although it is rare. In addition, the surface of early gastric SRCC is often covered by a normal mucosal epithelium and it is sometimes difficult to differentiate the lesion from the other common elevated lesions based on only endoscopic findings. Therefore, a biopsy should be mandatory when a single elevated lesion is seen in the stomach. In addition, it is worth noting that this lesion displayed a dense microvascular network with focal irregularity that is not typical microvascular pattern of early gastric cancer and was not described in the previous literature. Whether the dense microvascular network with focal irregularity is also an abnormal microvascular pattern of early gastric cancer needs plenty of evidence and investigations.

In conclusion, elevated gastric SRCC is rare. We herein present the endoscopic and pathohistologic features of an early elevated pure gastric SRCC. To our knowledge, this is the first report of the elevated early pure gastric SRRC and the description of its endoscopic and pathohistologic features, which contributes to promote the early detection of gastric SRCC and improve patients’ prognosis.

## Data availability statement

The original contributions presented in the study are included in the article/supplementary material. Further inquiries can be directed to the corresponding author.

## Ethics statement

The studies involving human participants were reviewed and approved by Ethics Committee of Zunyi Medical University. The patients/participants provided their written informed consent to participate in this study. Written informed consent was obtained from the individual(s) for the publication of any potentially identifiable images or data included in this article.

## Author contributions

The study design was performed by BT and LD. The review of patient data and critical comments were performed by LD, XW, and BT. LD and XW reviewed and described the pathohistologic and endoscopic findings. The manuscript was written by LD and BT. All authors read and approved the final manuscript.
